# Evaluation of PRDM10 gene rearrangement by immunohistochemistry and molecular methods in unclassifiable undifferentiated soft tissue tumors

**DOI:** 10.1007/s00795-025-00442-2

**Published:** 2025-06-25

**Authors:** Merve Aksin, Kivilcim Eren Ates, Akif Mirioglu, Tugba Toyran, Gulfiliz Gonlusen

**Affiliations:** 1 Department of Pathology, Faculty of Medicine, Cukurova University, Adana, Turkey; 2 Department of Orthopedics, Faculty of Medicine, Cukurova University, Adana, Turkey

**Keywords:** PRDM10, Undifferentiated pleomorphic sarcoma, Superficial CD34-positive fibroblastic tumor, Immunohistochemistry, Fluorescence in situ hybridization

## Abstract

Soft tissue sarcomas are heterogenous groups of tumors that show variable morphology as well as clinical behavior. Morphological features do not always directly reflect clinical behavior. Certain mesenchymal tumors exhibit an indolent clinical course. Among them are superficial CD34-positive fibroblastic tumors characterized by PRDM10 fusion. In our study, we aimed to detect PRDM10 gene rearrangement in superficial CD34-positive fibroblastic tumors and other pleomorphic sarcomas included in its differential diagnosis by immunohistochemistry and Fluorescence in situ hybridization. Totally, 33 cases were enrolled into this study. The results showed that two cases diagnosed as superficial CD34-positive fibroblastic tumor and two cases diagnosed as undifferentiated pleomorphic sarcoma have PRDM10 gene rearrangement. Immunohistochemically, not all rearranged tumors showed PRDM10 staining that suggests a low sensitivity of PRDM10 antibody. In conclusion, we suggested that PRDM10 gene rearrangement is not limited to superficial CD34-positive fibroblastic tumors; undifferentiated pleomorphic sarcomas may exhibit this molecular alteration and immunohistochemistry has lower sensitivity than fluorescence in situ hybridization.

## Introduction

Malignant soft tissue tumors are a highly heterogenous group of tumors with different appearances and subtypes, each with a widely varying prognosis. Undifferentiated pleomorphic sarcomas (UPS) are aggressive tumors, while the mimickers, low-grade myxofibrosarcomas (MFS), superficial CD34-positive fibroblastic tumors (SC34FT), myofibroblastic sarcomas, myxoinflammatory fibroblastic sarcomas (MIFS) and pleomorphic hyalinizing angiectatic tumors (PHAT) have a more benign course. [1].

PRDM proteins are a subgroup of the PR/SET transcription factor family, typically featuring the N-terminal PR domain. The PR domain is composed of homologous regions derived from PRDI-BF1 (positive regulatory domain I-binding factor 1) and RIZ1 (retinoblastoma-interacting zinc finger protein 1), and is subsequently followed by a variable number of Cys2–His2 (C2H2)-type zinc finger repeats. This PR domain, regulating gene expression, plays a pivotal role in the histone methylation pathway. The zinc finger repeats facilitate protein–protein and protein–nucleic acid interactions. Through the recognition of specific DNA sequences, transcription factors are able to bind to the promoter regions of target genes, thereby regulating gene expression. There are 19 identified members of the PRDM protein family in humans [2–4].

PRDM10, one of the least studied members of the PRDM family, is thought to act as a transcriptional cofactor [2, 3, 5]. SC34FT is a distinctive low-grade neoplasm of skin and subcutis that was first described in 2014 by Carter et al., which is recently renamed as PRDM10-rearranged soft tissue tumor in the 5th edition of WHO Classification of Tumors: Soft Tissue and Bone Tumors [1, 6]. It was previously described as a low-grade malignant fibrous histiocytoma or a low-grade sarcoma not otherwise classified [7]. Although SC34FT exhibits prominent nuclear pleomorphism similar to UPS, it differs by having extremely low mitotic rate and diffuse CD34 expression. The differential diagnosis of SCD34FT includes MIFS, PHAT, and myofibroblastic sarcoma, which display pleomorphic morphology. Yet, these are characterized by a low mitotic activity. MIFS is characterized by pleomorphic fibroblastic cells with macronucleoli in a myxohyaline background with a variably prominent inflammatory cell infiltrate. Myofibroblastic sarcoma is characterized by spindled tumor cells arranged in fascicles or show a storiform growth pattern. PHAT is characterized by ectatic blood vessels surrounded by pleomorphic, hemosiderin-rich spindle cells [1].

In this study, we aimed to detect PRDM10 gene rearrangement in superficial CD34-positive fibroblastic tumors and other pleomorphic sarcomas included in its differential diagnosis by immunohistochemistry and fluorescence in situ hybridization.

## Materials and methods

Our project, protocol number TTU-2022–14602, was approved by the Cukurova University, Faculty of Medicine, Non-Interventional Clinical Research Ethics Committee on November 5, 2021.

Inclusion criterion is the diagnoses of UPS, MFS, MIFS, myofibroblastic sarcoma, PHAT, and SC34FT. Exclusion criterion is: 1) tumors that had a mitotic score of 3 (mitotic count ≥ 20/10 HPF) and/or a necrosis score of 2 (tumor necrosis rate ≥ 50%) according to the FNCLCC grading system, 2) inadequate material for molecular analysis (less than 100 cells and/or 0.12 mm tumor area [8]).

Totally, 33 cases (15 UPS, 9 MFS, 2 MIFS, 3 myofibroblastic sarcoma, 2 PHAT, 2 SC34FT) were enrolled into this study.

Clinical and pathological information (including the immunohistochemical findings) was retrieved from the automation system and pathology reports.

Automated immunohistochemistry (IHC) using the primary rabbit polyclonal PRDM10 antibody (PA5-82,948, ThermoFisher Invitrogen, USA, 1:100) was performed on the selected blocks. Human duodenum tissues were used to optimize antibody dilution and antigen retrieval and served as positive controls.

Nuclear and/or cytoplasmic staining was considered positive for the PRDM10 antibody. The intensity and the extent of PRDM10 immunoreactivity were evaluated semi-quantitatively. Staining intensity was scored as 0 (no staining), + 1 (weak), + 2 (moderate), and + 3 (strong) compared to the control tissue. Extent of staining was scored as 0 (staining in < 5% of tumor cells), 1 (staining in 5–25% of tumor cells), 2 (staining in 26–50% of tumor cells), 3 (staining in 51–75% of tumor cells), and 4 (staining in > 75% of tumor cells).

PRDM10 probe (Empire Genomics, USA) was used for Fluorescence in situ hybridization (FISH). On each slide, 100 nuclei were counted, and a break-apart signal was considered present if the distance between red and green signals was at least twice the estimated signal diameter or if a single red signal was detected. More than 15 tumor cells with break-apart signals were considered positive for PRDM10 rearrangement.

Statistical analysis was performed on the population of 33 cases using SPSS (Statistical Package for the Social Sciences) 25.0 software, with a significance level of 0.05 for all tests. Chi-square test was used to compare categorical variables. Shapiro–Wilk test was employed to assess whether the parameters included in the study followed a normal distribution. For parameters that did not exhibit normal distribution, Mann–Whitney *U* test was applied.

## Results

Immunohistochemical staining for PRDM10 was positive in five cases (15.2%), except one for all of which were diagnosed as UPS (cases no 3, 4, 5, 6); remaining one case was diagnosed as SC34FT (case no 1) (an overview of the cases showing positivity by immunohistochemistry and/or FISH is presented in Table 1).

**Table 1:** PRDM10 Immunohistochemistry and/or FISH-positive cases

Cases	Original diagnosis	Extent of PRDM10 staining in tumor cells/immune score	Intensity of PRDM10 staining in tumor cells/immune score	FISH	CD34	Mitotic activity (per 10 HPFs)
Case 1 (Fig. 1)	SC34FT	90%/4	Strong nuclear and cytoplasmic/3	Positive (16%)	Diffuse positive	1
Case 2 (Fig. 2)	SC34FT	Negative/0	Negative/0	Positive (16%)	Diffuse positive	1
Case 3	UPS	20%/1	Strong nuclear and cytoplasmic/3	Negative (1%)	Negative	14
Case 4	UPS	10%/1	Intermediate cytoplasmic/2	Negative (1%)	Negative	14
Case 5	UPS	10%/1	Weak cytoplasmic/1	Negative (1%)	Negative	12
Case 6	UPS	5%/1	Weak cytoplasmic/1	Negative (1%)	Negative	15
Case 7	UPS	Negative/0	Negative/0	Positive (17%)	Negative	13
Case 8	UPS	Negative/0	Negative/0	Positive (19%)	Negative	12

One SC34FT (case no 1) and one UPS (case no 3) case exhibited strong intensity in both nuclear and cytoplasmic staining. One UPS (case no 4) showed moderate intensity in cytoplasmic staining, and remaining two UPS (case nos. 5 and 6) cases showed weak cytoplasmic staining.

The SC34FT case (case no 1) showed staining in more than 75% of cells, while the other four UPS cases (case no 3, 4, 5, 6) showed staining in 5–25% of cells. The total IHC scores were shown in Table 1 respectively.

There were no statistical significant differences between age, gender, tumor depth, tumor size, local recurrence, metastasis, survival, and CD34 immunoreactivity based on the presence of PRDM10 staining (*p* > 0.05) (clinicopathological findings are summarized in Table 2).

**Table 2:** Clinical features of the immunohistochemical and/or FISH-positive cases

Cases	Age	Sex	Tumor site	Tumor depth	Tumor size (cm)	Local recurrence/distant metastasis (follow-up (mo))	Survival
Case 1	37	F	Lower extremity	Superficial	11	−/− (21)	Alive
Case 2	43	F	Lower extremity	Superficial	3	−/− (78)	Alive
Case 3	81	M	Lower extremity	Deep	18	−/− (4)	Ex
Case 4	46	M	Lower extremity	N/A	N/A	+/− (93)	Alive
Case 5	74	F	Upper extremity	Deep	13	−/+ * (14)	Ex
Case 6	33	M	Trunk	Deep	12	N/A/N/A	N/A
Case 7	72	F	Lower extremity	Superficial	4	+/+ ** (44)	Alive
Case 8	68	F	Head and neck	Superficial	4	−/− (3)	Ex

*Lung metastasis, **Upper extremity metastasis

**Fig. 1:**
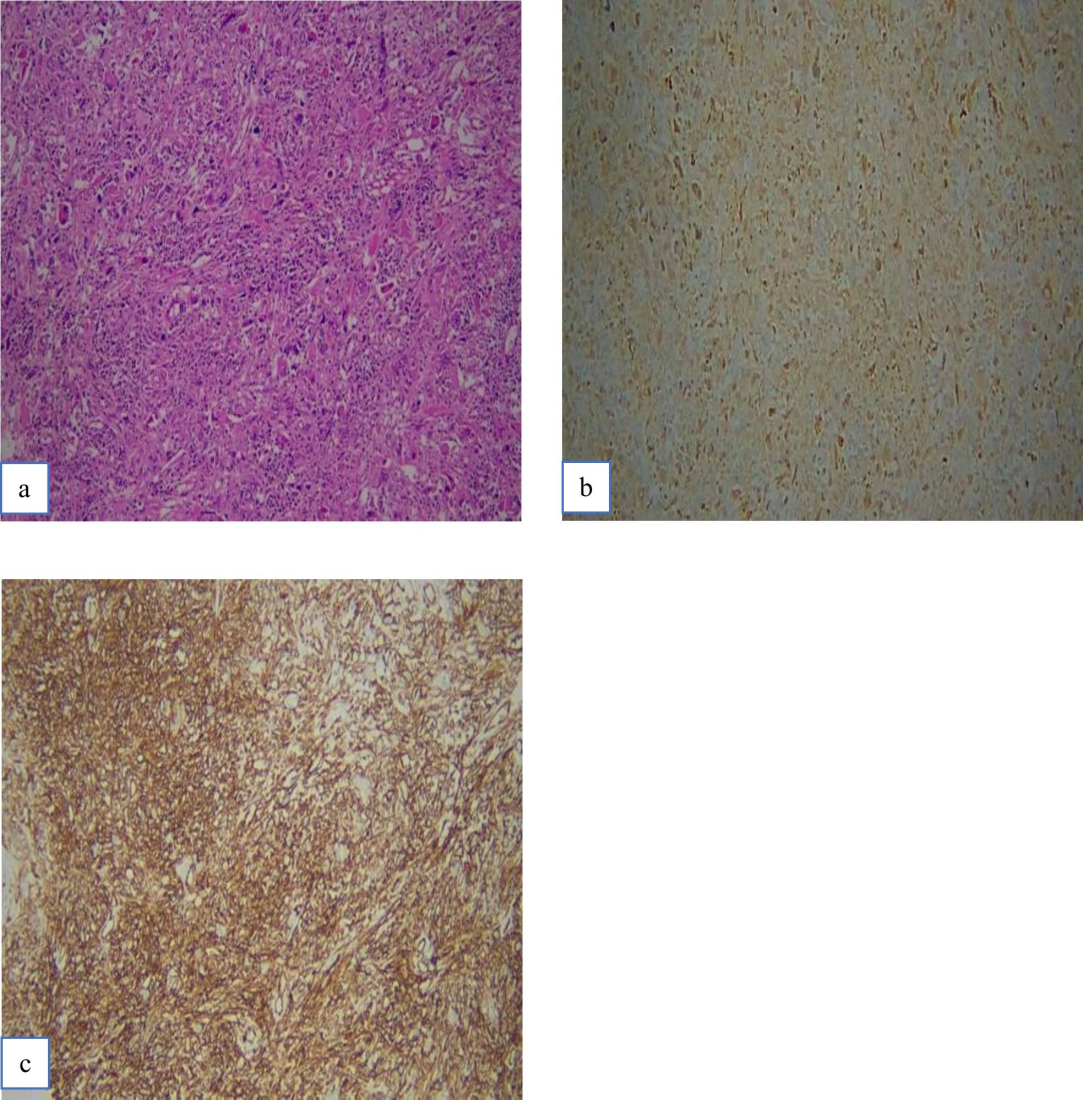
Case no 1. **a** H&E. **b** PRDM10 positivity. **c** CD34 positivity

**Fig. 2:**
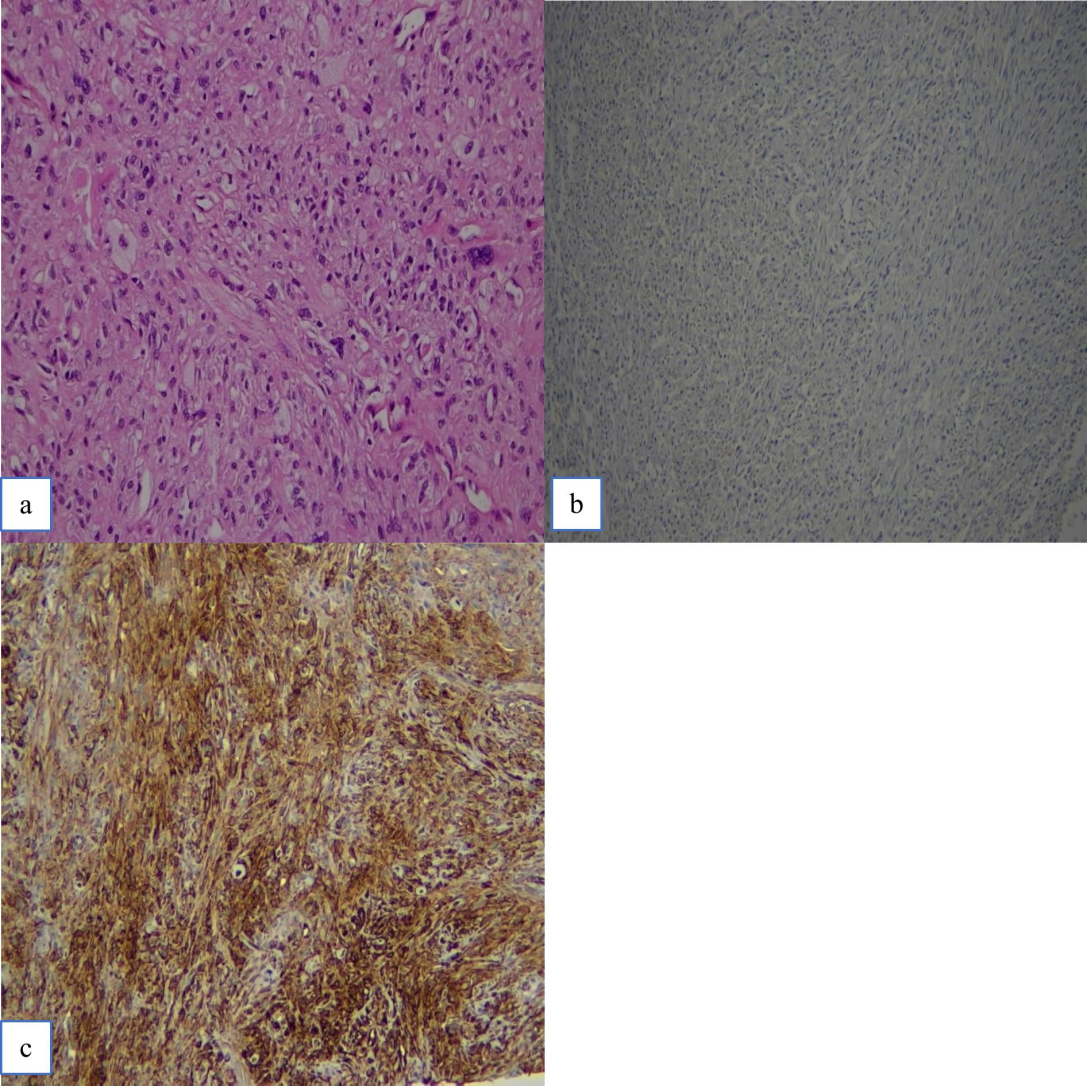
Case no 2. **a** H&E. **b** PRDM10 negativity. **c** CD34 positivity

Break-apart signals were detected in four (12.1%) of the 33 cases by FISH analysis (Table 1). Two of these four cases were diagnosed as UPS (case nos. 7 and 8); remaining two cases were as SC34FT (case nos. 1 and 2). The first case of SC34FT (case no. 1) also showed a positive reaction with the PRDM10 antibody immunohistochemically, in which strong nuclear and cytoplasmic positivity was observed in 90% of tumor cells (Fig. 1). The other SC34FT case (case no. 2) (Fig. 2) and the other two UPS cases were negative with the PRDM10 antibody.

Although FISH-positive cases were observed higher in female and elderly patients, the difference was not statistically significant (*p* > 0.05). Notably, FISH positivity was frequently observed in superficially located four tumors (p: 0.06). However, statistically, no significant differences were found in tumor depth, tumor size, local recurrence, metastasis, survival, and CD34 immunoreactivity (*p* > 0.05) (Table 2).

## Discussion

The importance of this study is to apply both PRDM10 IHC and FISH to all cases to detect PRDM10 gene rearrangement, and it is the first study to detect FISH positivity in a tumor group other than SC34FTs.

SC34FT is a rare soft-tissue neoplasm that is previously classified into UPS [7]. Recent molecular developments exhibited PRDM10 rearrangement in SC34FT [9–12]. The discovery of PRDM10 in soft-tissue tumors in 2015 offered a new era for investigation [13]. In that study, PRDM10 fusion was detected in three UPS cases. However, significant pleomorphism was detected, and mitotic activity was remarkably low (less than 1 per 10 HPFs) in these three tumors. This finding suggests that these tumors might be low-grade soft tissue tumors in contrast to pleomorphic sarcomas. Subsequent studies revealed that tumors previously described as low-grade UPS were, in fact, SC34FT [9–11]. Previously, Puls et al. was described PRDM10-rearranged tumors as a separate entity, but in 2022, the same group revisited PRDM10-rearranged tumors to SC34FT [9, 11].

The corresponding antibodies of molecular alterations are more useful and cost-effective. However, immunohistochemical studies haven’t reached a consensus on which staining pattern (nuclear and/or cytoplasmic) is significant for the PRDM10-rearranged tumors. Puls et al. considered only nuclear staining is significant, whereas Sugita et al. showed the importance of cytoplasmic staining as well in PRDM10- rearranged tumors [9, 14]. Furthermore, PRDM10 IHC has been found positive not only in low-grade malignant or borderline tumors but also in many high-grade soft tissue tumors [15].

In our study, we considered both nuclear and cytoplasmic staining as positive. While all the positive cases were highly cellular and pleomorphic, SC34FT case (case no. 1) showed widespread PRDM10 positivity (90% of cells stained). This case, divergent from the other four PRDM10 positive cases, exhibited exceptionally low mitotic activity (1–2 per 50 HPFs) and no tumor necrosis. It was also the only positive case for both PRDM10 (FISH) and CD34 (IHC).

The other FISH-positive case exhibited diffuse positivity with CD34 and diagnosed as SC34FT (case no. 2). This case lacked PRDM10 staining that suggests a low sensitivity of the PRDM10 antibody. Moreover, the intensity and the extent of staining should be considered together.

Studies examining PRDM10 IHC are also insufficient in determining a precise threshold for the extent of staining. In our study, the case no. 1 with high-intensity, widespread (90% of cells) nuclear staining was definitely considered positive through correlation with FISH. However, in the case no. 3 with high intensity, but only 20% of tumor cells stained, it was concluded that a higher threshold value might be necessary. Therefore, a high intensity and a high extent of staining seem meaningful; however, a more accurate threshold is needed for the extent of tumor cell staining. We believe moderate-/low-intensity staining or low proportions of stained cells should be further confirmed with molecular techniques such as FISH. In the four UPS cases which were immunohistochemically PRDM10-positive, PRDM10 gene rearrangement was not detected by FISH.

Variations in the sensitivity and the specificity of PRDM10 IHC have led to the search for new immunomarkers. The SynCAM3 (CADM3) immunomarker has been found to be highly useful in detecting PRDM10-rearranged tumors compared to PRDM10 immunomarker. [10, 11, 14, 16].

FISH is an ancillary method used alongside IHC in the diagnosis of sarcomas. However, the threshold values to determine gene rearrangements vary between individual probes and laboratories [17, 18]. The detection of PRDM10 gene rearrangement involves the use of break-apart probes targeting the breakpoint in the gene. In our study, we accepted a threshold value of 15% for signal separation in FISH. Various studies have so far chosen a threshold value, ranging from 10 to 20% of tumor cells [9, 11, 14]. The lack of standardization for the threshold value could pose an issue when there are borderline values.

In our study, we detected FISH positivity in 2 SC34FT and 2 UPS cases, demonstrating that the PRDM10-rearranged soft tissue-tumor spectrum is not limited to SC34FTs alone. Interestingly, IHC positivity was observed only in one SC34FT case in these four. PRDM10 FISH positivity is not specific for SC34FTs, but may serve as a supportive finding for diagnosis.

In conclusion, SC34FTs have PRDM10 gene rearrangement, superficial location, low mitotic activity, no necrosis, and CD34 positivity, but it should be kept in mind that PRDM10 immunohistochemistry may be negative in these tumors. UPS, vice versa, exhibit PRDM10 gene rearrangement as well as positive staining.

This study contains limited numbers of cases. Therefore, it should be enlarged with multicenter studies. A combined approach using IHC, FISH, RNA sequencing, and RT-PCR methods is likely to yield the most reliable results. 

## Data Availability

All data underlying the results are available as part of the article and no additional source data are required.

## References

[1] WCoTE B (2020) Soft tissue and bone tumours WHO classification of tumours, vol 3, 5th edn. IARC Publications, Lyon

[2] Di Zazzo E, De Rosa C, Abbondanza C, Moncharmont B (2013) PRDM Proteins: molecular mechanisms in signal transduction and transcriptional regulation. Biology (Basel) 2(1):107–141 24832654 10.3390/biology2010107 PMC4009873

[3] Mzoughi S, Tan YX, Low D, Guccione E (2016) The role of PRDMs in cancer: one family, two sides. Curr Opin Genet Dev 36:83–91 27153352 10.1016/j.gde.2016.03.009

[4] Sorrentino A, Rienzo M, Ciccodicola A, Casamassimi A, Abbondanza C (2018) Human PRDM2: structure, function and pathophysiology. Biochim Biophys Acta Gene Regul Mech 1861:657–671 10.1016/j.bbagrm.2018.06.002 29883756

[5] Park JA, Kim KC (2010) Expression patterns of PRDM10 during mouse embryonic development. BMB Rep 43(1):29–33 20132732 10.5483/bmbrep.2010.43.1.029

[6] Carter JM, Weiss SW, Linos K, DiCaudo DJ, Folpe AL (2014) Superficial CD34-positive fibroblastic tumor: report of 18 cases of a distinctive low-grade mesenchymal neoplasm of intermediate (borderline) malignancy. Mod Pathol 27(2):294–302 23887307 10.1038/modpathol.2013.139

[7] Sood N, Khandelia BK (2017) Superficial CD34-positive fibroblastic tumor: a new entity; case report and review of literature. Indian J Pathol Microbiol 60(3):377–380 28937375 10.4103/IJPM.IJPM_589_16

[8] Scarpino S, Pulcini F, Di Napoli A, Giubettini M, Ruco L (2015) EGFR mutation testing in pulmonary adenocarcinoma: evaluation of tumor cell number and tumor percent in paraffin sections of 120 small biopsies. Lung Cancer 87(1):8–13 25468201 10.1016/j.lungcan.2014.10.012

[9] Puls F, Pillay N, Fagman H, Palin-Masreliez A, Amary F, Hansson M et al (2019) PRDM10-rearranged soft tissue tumor: a clinicopathologic study of 9 cases. Am J Surg Pathol 43(4):504–513 30570551 10.1097/PAS.0000000000001207

[10] Perret R, Michal M, Carr RA, Velasco V, Švajdler M, Karanian M et al (2021) Superficial CD34-positive fibroblastic tumor and PRDM10-rearranged soft tissue tumor are overlapping entities: a comprehensive study of 20 cases. Histopathology 79(5):810–825 34121219 10.1111/his.14429

[11] Puls F, Carter JM, Pillay N, McCulloch TA, Sumathi VP, Rissler P et al (2022) Overlapping morphological, immunohistochemical and genetic features of superficial CD34-positive fibroblastic tumor and PRDM10-rearranged soft tissue tumor. Mod Pathol 35(6):767–776 34969957 10.1038/s41379-021-00991-8

[12] Zhao M, Yin X, He H, Fan Y, Ru G, Meng X (2023) Recurrent PRDM10 fusions in superficial CD34-positive fibroblastic tumors : a clinicopathologic and molecular study of 10 additional cases of an emerging novel entity. Am J Clin Pathol 159(4):367–378 36812381 10.1093/ajcp/aqac171

[13] Hofvander J, Tayebwa J, Nilsson J, Magnusson L, Brosjö O, Larsson O et al (2015) Recurrent PRDM10 gene fusions in undifferentiated pleomorphic sarcoma. Clin Cancer Res 21(4):864–869 25516889 10.1158/1078-0432.CCR-14-2399

[14] Sugita S, Takenami T, Kido T, Aoyama T, Hosaka M, Segawa K et al (2023) Usefulness of SynCAM3 and cyclin D1 immunohistochemistry in distinguishing superficial CD34-positive fibroblastic tumor from its histological mimics. Med Mol Morphol 56(1):69–77 36344703 10.1007/s00795-022-00341-w

[15] Mistik O, Sayar H (2022) Immunohistochemical positive regulatory domain member 10 expression in soft tissue sarcomas. Pol J Pathol 73(3):223–232 36734437 10.5114/pjp.2022.124490

[16] Hofvander J, Puls F, Pillay N, Steele CD, Flanagan AM, Magnusson L et al (2019) Undifferentiated pleomorphic sarcomas with PRDM10 fusions have a distinct gene expression profile. J Pathol 249(4):425–434 31313299 10.1002/path.5326

[17] Sugita S, Hasegawa T (2017) Practical use and utility of fluorescence in situ hybridization in the pathological diagnosis of soft tissue and bone tumors. J Orthop Sci 22(4):601–612 28274512 10.1016/j.jos.2017.02.004

[18] Papp G, Mihály D, Sápi Z (2017) Unusual signal patterns of break-apart FISH probes used in the diagnosis of soft tissue sarcomas. Pathol Oncol Res 23(4):863–871 28108880 10.1007/s12253-017-0200-z

